# A mathematical model for the auxetic response of liquid crystal elastomers

**DOI:** 10.1098/rsta.2021.0326

**Published:** 2022-10-17

**Authors:** L. Angela Mihai, Devesh Mistry, Thomas Raistrick, Helen F. Gleeson, Alain Goriely

**Affiliations:** ^1^ School of Mathematics, Cardiff University, Cardiff CF24 4AG, UK; ^2^ School of Physics and Astronomy, University of Leeds, Leeds LS2 9JT, UK; ^3^ Mathematical Institute, University of Oxford, Oxford OX2 6GG, UK

**Keywords:** liquid crystals, elastomers, auxetic behaviour, finite deformation, mathematical modelling

## Abstract

We develop a mathematical model that builds on the surprising nonlinear mechanical response observed in recent experiments on nematic liquid crystal elastomers. Namely, under uniaxial tensile loads, the material, rather than thinning in the perpendicular directions, becomes thicker in one direction for a sufficiently large strain, while its volume remains unchanged. Motivated by this unusual large-strain auxetic behaviour, we model the material using an Ogden-type strain-energy function and calibrate its parameters to available datasets. We show that Ogden strain-energy functions are particularly suitable for modelling nematic elastomers because of their mathematical simplicity and their clear formulation in terms of the principal stretches, which have a direct kinematic interpretation.

This article is part of the theme issue ‘The Ogden model of rubber mechanics: Fifty years of impact on nonlinear elasticity’.

## 1. Introduction

Quasi-static uniaxial tensile tests on cat skin suggest that the reorientation of dermal fibres causes an initial increase in the skin thickness, while the material volume may either increase or decrease [1] (see also the review in [2]). By contrast, many natural and synthetic materials tend to become thinner in any direction perpendicular to the tensile load independently of volume changes.

**Figure 1.**
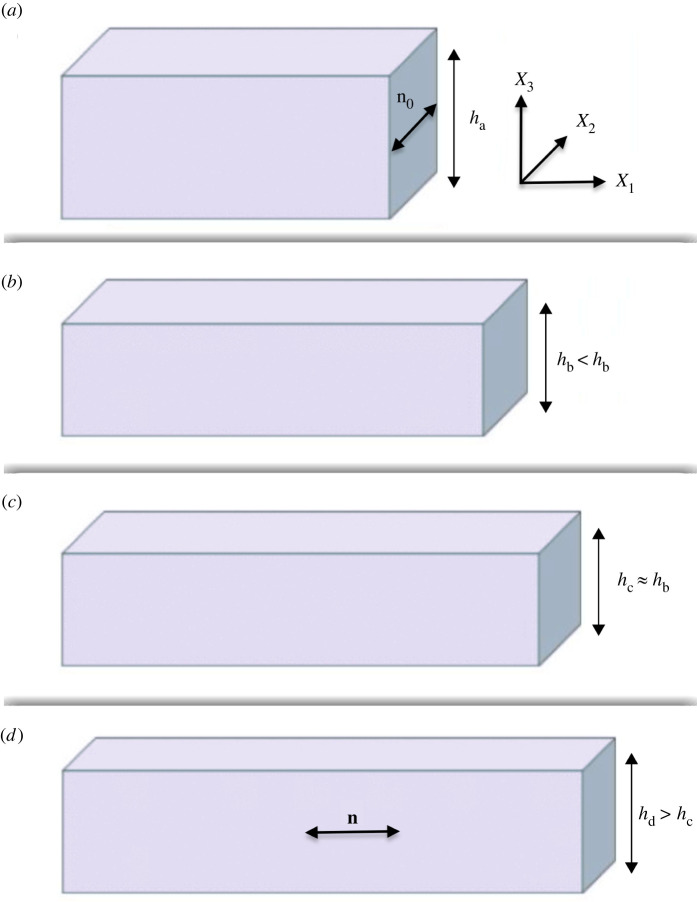
When the reference auxetic LCE sample (*a*) is stretched horizontally (in the $X_{1}$ direction), its volume remains unchanged, while its thickness first decreases, $h_{\text{b}}<h_{\text{a}}$ (*b*), then is preserved almost unchanged $h_{\text{c}}\approx h_{\text{b}}$ (*c*), then increases again $h_{\text{d}}>h_{\text{c}}$ (*d*). In this LCE, the nematic director n is initially aligned in the second direction, along ${\text{n}}_{0}$, then rotates by π/2 to become parallel to the applied force. (Online version in colour.)

Recently, a novel nematic liquid crystal elastomer (LCE) was shown to exhibit similar behaviour, namely, the thickness of a stretched material sample increases at sufficiently large strain while the material volume remains unchanged [3–6] (figure 1). When viewed as a two-dimensional system, this auxetic effect (from the Greek word αὔξησις for ‘growth’ or ‘increase’) coincides with an apparent sharp rotation, by π/2, of the average nematic alignment direction. However, in three dimensions, the sharp director rotation is accompanied by a gradual decrease and then increase in uniaxial orientational order coupled with the emergence and subsequent loss of biaxial symmetry [6].

This is different from the mechanical response of most other nematic LCEs, with a continuous rotation of the director and a constant uniaxial order parameter, where shear striping patterns can develop [7–13]. The so-called *soft elasticity* phenomenon where alternating shear stripes occur at very low stress has been studied extensively. Its theoretical explanation is that, for these materials, the energy is minimized by a state exhibiting a microstructure of many homogeneously deformed parts [8,14–22].

In this paper, we present a mathematical model that builds on the auxeticity of a novel nematic LCE, which deforms with a sharp rotation of the director at large strains, within the theoretical framework of finite elasticity [23–25]. Specifically, a phenomenological Ogden-type strain-energy function is adopted that matches the available experimental data with a relatively small number of terms. In §2, we introduce and explain the chosen strain-energy function. This is followed, in §3, by the calibration of model parameters to experimental data from uniaxial tensile tests. Directions for future investigation are outlined in the concluding remarks.

## 2. A continuum model for auxetic liquid crystal elastomers

Nematic LCEs are cross-linked networks of polymeric chains containing liquid crystal mesogens [26–28]. Their manufactured molecular structure then renders them capable of large reversible deformations and makes them highly responsive to external stimuli, such as heat, light, solvents and electric or magnetic fields [29–45].

Because of their intrinsic similarities with conventional rubber, suitable descriptions of LCEs can be achieved by adapting existing hyperelastic models for rubber-like solids. For example, a simple continuum model for ideal monodomains, where the director is uniformly aligned throughout the material, is the so-called neoclassical model [46–48]. This is based on the molecular network theory of rubber [49] where the parameters of the neo-Hookean-type strain-energy density is derived from macroscopic shape changes at small strain or through statistical averaging at microscopic scale [28,50]. Phenomenological models based on other hyperelastic strain-energy functions (e.g. Mooney–Rivlin, Gent, Ogden) that capture the nonlinear elastic behaviour at large strains have also been developed [16,51,52].

To model an incompressible nematic LCE, we first introduce the following isotropic elastic strain-energy function [21,22] (see also [14,17–19,53]),

2.1 $$W^{\left(el\right)}(\text{F},\text{Q},\text{n})=W^{\left(1\right)}(\text{F})+W^{\left(2\right)}({\text{G}}^{-1}\text{F}{\text{G}}_{0}),$$

where F denotes the deformation gradient from the reference cross-linking state, satisfying detF=1, and n is a unit vector for the (localized) direction of uniaxial nematic alignment in the present configuration, referred to as the *director*. We denote by ${\text{n}}_{0}$ the reference orientation of the local director corresponding to the cross-linking state. On the right-hand side of equation (2.1), the first term is the strain-energy density associated with the overall macroscopic deformation, and the second term represents the strain-energy density of the polymer microstructure, with ${\text{G}}_{0}$ and G denoting the ‘natural’ (or ‘spontaneous’) deformation tensor in the reference and current configuration, respectively. These tensors are assumed to satisfy the following relations [54] (see also [28], ch. 3):

2.2 $${\text{G}}_{0}^{2}=c_{0}(\text{I}+2{\text{Q}}_{0})\;\mathrm{and}\;{\text{G}}^{2}=c(\text{I}+2\text{Q}),$$

where $c_{0}$ and *c* represent the effective step length of the polymeric chain, I=diag(1,1,1) is the tensor identity, with diag(⋅,⋅,⋅) denoting the diagonal second-order tensor, and ${\text{Q}}_{0}$ and Q are the symmetric traceless order parameter tensors ([28], pp. 48–49). The macroscopic tensor parameter describes orientational order in nematic liquid crystals [55].

Next, we define the nematic strain-energy function given by the Landau-de Gennes expansion in powers of the tensor order parameter [28, p. 15],

2.3 $$W^{\left(lc\right)}(\text{Q})=\frac{1}{3}A\mathrm{tr}(\text{Q}\text{Q})-\frac{4}{9}B\mathrm{tr}(\text{Q}\text{Q}\text{Q})+\frac{2}{9}C\mathrm{tr}(\text{Q}\text{Q}\text{Q}\text{Q})+\cdots .$$

For incompressible nematic elastomers subjected to uniaxial stretches, the contribution given by the above nematic function to the total strain-energy density was originally analysed in [54], and more recently in [56–58]. In particular, it was demonstrated that there are significant differences between the mechanical behaviour of real nematic solids and those of ideal LCE models are described only by an isotropic elastic strain energy.

Using the strain-energy functions described by (2.1) and (2.3), the composite LCE model function then takes the form

2.4 $$W^{\left(\mathrm{lce}\right)}(\text{F},\text{Q},\text{n})=W^{\left(el\right)}(\text{F},\text{Q},\text{n})+W^{\left(lc\right)}(\text{Q}),$$

where F, Q and n are mutually independent variables.

The associated Cauchy stress tensor represents the internal force acting within the deformed solid on a unit of deformed area and is equal to

2.5 $${\text{T}}^{\left(\mathrm{lce}\right)}=\frac{\partial W^{\left(\mathrm{lce}\right)}}{\partial \text{F}}{\text{F}}^{T}-p\text{I},$$

where p is the Lagrange multiplier for the incompressibility constraint detF=1, and the upper case T denotes the transpose.

The corresponding first Piola–Kirchhoff stress tensor, representing the internal force acting within the deformed body on an area element which in its reference state was one unit of area, is

2.6 $${\text{P}}^{\left(\mathrm{lce}\right)}={\text{T}}^{\left(\mathrm{lce}\right)}{\text{F}}^{-T},$$

where −T denotes the inverse transpose. The tensor ${\text{P}}^{(\mathrm{lce})T}$ is known as the nominal stress tensor [24, pp. 152–153].

For the elastic components of the LCE model, we use an Ogden-type strain-energy density function [59], as follows:

2.7 $$W^{\left(1\right)}(\lambda_{1},\lambda_{2},\lambda_{3})=\sum_{j=1}^{m}\frac{c_{j}^{\left(1\right)}}{2{\left(p_{j}^{\left(1\right)}\right)}^{2}}\left(\lambda_{1}^{2p_{j}^{\left(1\right)}}+\lambda_{2}^{2p_{j}^{\left(1\right)}}+\lambda_{3}^{2p_{j}^{\left(1\right)}}-3\right),$$

where ${\left\{c_{j}^{\left(1\right)}\right\}}_{j=1,\ldots ,m}$ and ${\left\{p_{j}^{\left(1\right)}\right\}}_{j=1,\ldots ,m}$ are constants independent of the deformation, and $\left\{\lambda_{1}^{2},\lambda_{2}^{2},\lambda_{3}^{2}\right\}$ are the eigenvalues of the tensor ${\text{F}}^{T}\text{F}$, such that $\lambda_{1}\lambda_{2}\lambda_{3}=1$, and

2.8 $$W^{\left(2\right)}(\alpha_{1},\alpha_{2},\alpha_{3})=\sum_{j=1}^{n}\frac{c_{j}^{\left(2\right)}}{2{\left(p_{j}^{\left(2\right)}\right)}^{2}}\left(\alpha_{1}^{2p_{j}^{\left(2\right)}}+\alpha_{2}^{2p_{j}^{\left(2\right)}}+\alpha_{3}^{2p_{j}^{\left(2\right)}}-3\right),$$

where ${\left\{c_{j}^{\left(2\right)}\right\}}_{j=1,\ldots ,n}$ and ${\left\{p_{j}^{\left(2\right)}\right\}}_{j=1,\ldots ,n}$ are constants independent of the deformation, and $\left\{\alpha_{1}^{2},\alpha_{2}^{2},\alpha_{3}^{2}\right\}$ are the eigenvalues of the elastic Cauchy–Green tensor ${\text{A}}^{T}\text{A}$, such that $\alpha_{1}\alpha_{2}\alpha_{3}=1$, with the local elastic deformation tensor $\text{A}={\text{G}}^{-1}\text{F}{\text{G}}_{0}$.

The composite model defined by (2.4) then takes the form

2.9 $$W^{\left(\mathrm{lce}\right)}(\lambda_{1},\lambda_{2},\lambda_{3},\text{Q})=W^{\left(1\right)}(\lambda_{1},\lambda_{2},\lambda_{3})+W^{\left(2\right)}(\alpha_{1},\alpha_{2},\alpha_{3})+W^{\left(lc\right)}(\text{Q}).$$

For this model, the principal Cauchy stresses are equal to

2.10 $$T_{i}^{\left(\mathrm{lce}\right)}=\frac{\partial W^{\left(\mathrm{lce}\right)}}{\partial \lambda_{i}}\lambda_{i}-p,\;i=1,2,3.$$

The associated first Piola–Kirchhoff stresses are

2.11 $$P_{i}^{\left(\mathrm{lce}\right)}=T_{i}^{\left(\mathrm{lce}\right)}\lambda_{i}^{-1},\;i=1,2,3.$$

In particular, if $c_{j}^{\left(1\right)}=0$, for all j=1,…,m, and $p_{1}^{\left(2\right)}=1$ and $p_{j}^{\left(2\right)}=0$ for all j=2,…,n, while $W^{\left(lc\right)}(\text{Q})$ is omitted, then the strain-energy function described by (2.9) reduces to the neoclassical model. However, for suitable parameter values, this composite function can capture nonlinear large-strain effects, as demonstrated next.

## 3. Representation of experimental data for uniaxial tests

Many nematic LCEs have uniaxial symmetry, given by the nematic director n [60–62]. However, strain-induced biaxial nematic order, with a secondary axis of symmetry in a plane orthogonal to n, is also possible ([28], Sec 6.6; for a summary of nematic order see also [28], Sec. 2.2). In [3–6], experimental observations for a nematic LCE exhibiting auxetic effects when subject to a uniaxial tensile force were reported where biaxial symmetry emerges. We choose a Cartesian system of coordinates $\left(X_{1},X_{2},X_{3}\right)$ in which the tensile force is applied in the first, or longitudinal, direction, while the second direction is along the reference director $n_{0}$, and designate the third direction as the direction of thickness (figure 1). Then, according to the experimental results, if the material sample is stretched longitudinally, its volume remains unchanged, but its thickness first decreases, then is preserved almost constant for a tensile range, then increases again, while the director suddenly rotates to align in the direction of the applied force.

**Figure 2.**
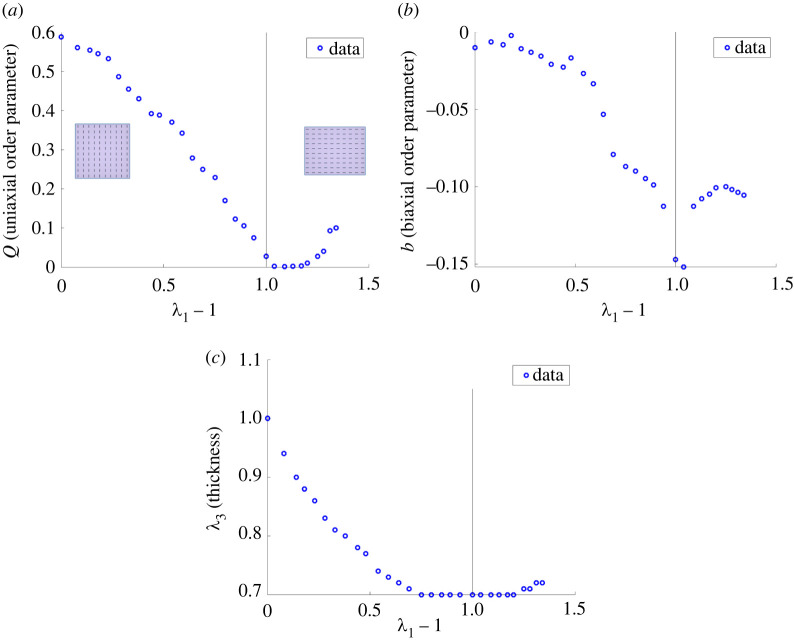
Experimental data recorded in table 2 for: (*a*) the uniaxial scalar order parameter Q; (*b*) the biaxial scalar order parameter b; (*c*) the sample thickness $\lambda_{3}$ versus the longitudinal strain $\lambda_{1}-1$. The nematic director is oriented in the second direction until a critical strain is reached and the director suddenly aligns in the first direction, i.e. parallel to the applied tensile load. In each plot, the vertical line is drawn at the critical strain where the director suddenly rotates by π/2. (Online version in colour.)

**Figure 3.**
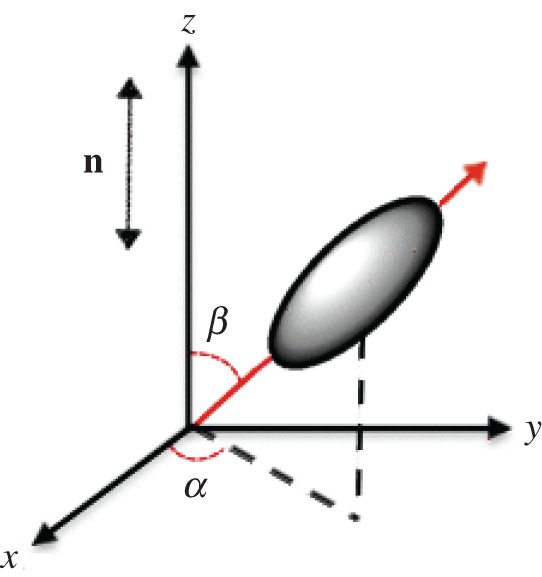
Schematic of the molecular frame with respect to the director frame used in the derivation of uniaxial order parameter $Q=\langle (3/2)\cos^{2}\beta -(1/2)\rangle$ and biaxial order parameter $b=(3/2)\langle \sin^{2}\beta \cos(2\alpha )\rangle$, where ⟨⋅⟩ denotes average value over mesogen angles α and β. (Online version in colour.)

In this section, we calibrate a model function of the form given by (2.9) to the experimental data for $\left(\lambda_{1},\theta ,Q,b,\lambda_{3},P_{1}^{\left(\mathrm{lce}\right)}\right)$ recorded in table 2 (see appendix A). These data values are slightly idealized compared to those reported in [6], in the sense that the angle θ for the director orientation remains equal to π/2 until a critical extension is reached, then it becomes 0, i.e. it is assumed that the director rotates by π/2 instantly. Note that, in figure 2*c*, the sample thickness first decreases, then increases at a critical large strain. Simultaneously, in figure 2*a*, the magnitude of the uniaxial order parameter decreases until the same critical strain is reached, then increases, while in figure 2*b*, the magnitude of the biaxial order parameter is larger around the critical strain. We set $Q=P_{200}$ and $b=6P_{220}$, where $P_{200}$ and $P_{220}$ are determined via Raman spectroscopy in [6]. A schematic of the molecular frame with respect to the director frame used in the derivation of order parameters is shown in figure 3. For these parameters, Q=1 corresponds to perfect nematic order while Q=0 is when mesogens are randomly oriented, and if b=0, then the system reduces to the uniaxial case.

**Table 1.** Parameters of the strain-energy function described by (3.7) calibrated to experimental data recorded in table 2. Numerical and experimental results are compared in figures 4 and 5. At small strain, the computed elastic modulus is equal to $E=3(c_{1}^{\left(1\right)}+c_{2}^{\left(1\right)}+c_{1}^{\left(2\right)})=8.2850$ (MPa) [67].

model function	calibrated parameters
$W^{\left(1\right)}$ defined by (2.7)	$c_{1}^{\left(1\right)}=-0.0771$, $p_{1}^{\left(1\right)}=2.5429$, $c_{2}^{\left(1\right)}=2.8372$, $p_{2}^{\left(1\right)}=-1.5690$
$W^{\left(2\right)}$ defined by (2.8)	$c_{1}^{\left(2\right)}=0.0015$, $p_{1}^{\left(2\right)}=0.7411$
$W^{\left(lc\right)}$ defined by (2.3)	A=0.0235, B=0.0008, C=−0.0268

Setting the nematic director in the reference and current configuration as ${\text{n}}_{0}={\left[0,1,0\right]}^{T}$ and $\text{n}={\left[\cos\theta ,\sin\theta ,0\right]}^{T}$, respectively, where θ∈[0,π/2], the deformation gradient takes the form

3.1 $$\text{F}=\mathrm{diag}(\lambda_{1},\lambda_{2},\lambda_{3}),$$

where $\lambda_{1}\lambda_{2}\lambda_{3}=1$. In fact, for the uniaxial deformation under consideration, all tensors involved share the same principal directions, and thus, are all diagonal.

In the reference configuration, the LCE is uniaxial, and the order parameter tensor is equal to [28, p. 14]:

3.2 $${\text{Q}}_{0}=\mathrm{diag}\left(-\frac{Q_{0}}{2},Q_{0},-\frac{Q_{0}}{2}\right),$$

where $Q_{0}$ is the scalar order parameter.

In the deformed configuration, when biaxiality emerges [28, p. 15]:

— If θ=π/2, then the order parameter tensor takes the form
  3.3 $$\text{Q}=\mathrm{diag}\left(-\frac{Q-b}{2},Q,-\frac{Q+b}{2}\right),$$
— If θ=0, then
  3.4 $$\text{Q}=\mathrm{diag}\left(Q,-\frac{Q-b}{2},-\frac{Q+b}{2}\right),$$

where Q and b are the uniaxial and biaxial scalar order parameters, respectively.

For the elastic Cauchy–Green tensor ${\text{A}}^{T}\text{A}$, by (2.2)

— If θ=π/2, then
  3.5 $$\begin{array}{rl} \alpha_{1}^{2} & \;={\left[\frac{\det\left(I+2Q\right)}{\det\left(I+2Q_{0}\right)}\right]}^{1/3}\frac{1-Q_{0}}{1-(Q-b)}\lambda_{1}^{2},\;\alpha_{2}^{2}={\left[\frac{\det\left(I+2Q\right)}{\det\left(I+2Q_{0}\right)}\right]}^{1/3}\frac{1+2Q_{0}}{1+2Q}\lambda_{2}^{2},\\ \alpha_{3}^{2} & \;={\left[\frac{\det\left(I+2Q\right)}{\det\left(I+2Q_{0}\right)}\right]}^{1/3}\frac{1-Q_{0}}{1-(Q+b)}\lambda_{3}^{2}; \end{array}$$
— If θ=0, then
  3.6 $$\begin{array}{rl} \alpha_{1}^{2} & \;={\left[\frac{\det\left(I+2Q\right)}{\det\left(I+2Q_{0}\right)}\right]}^{1/3}\frac{1-Q_{0}}{1+2Q}\lambda_{1}^{2},\;\alpha_{2}^{2}={\left[\frac{\det\left(I+2Q\right)}{\det\left(I+2Q_{0}\right)}\right]}^{1/3}\frac{1+2Q_{0}}{1-(Q-b)}\lambda_{2}^{2},\\ \alpha_{3}^{2} & \;={\left[\frac{\det\left(I+2Q\right)}{\det\left(I+2Q_{0}\right)}\right]}^{1/3}\frac{1-Q_{0}}{1-(Q+b)}\lambda_{3}^{2}. \end{array}$$

The general model described by (2.9) can capture, in theory, both the nonlinear elasticity and the nematic properties of the LCE material. In practice, many specific models of this form that differ in number of terms can be obtained that reasonably approximate the data. We apply Occam’s principle [63] and select a model with a relatively small number of terms, since simpler models are more likely to be used even if their approximation of the observed phenomena are not the best, as advocated in [64]. The chosen model function takes the form

3.7 $$\begin{array}{rl} W^{\left(\mathrm{lce}\right)} & \;=\frac{c_{1}^{\left(1\right)}}{2{\left(p_{1}^{\left(1\right)}\right)}^{2}}\left(\lambda_{1}^{2p_{1}^{\left(1\right)}}+\lambda_{2}^{2p_{1}^{\left(1\right)}}+\lambda_{3}^{2p_{1}^{\left(1\right)}}-3\right)+\frac{c_{2}^{\left(1\right)}}{2{\left(p_{2}^{\left(1\right)}\right)}^{2}}\left(\lambda_{1}^{2p_{2}^{\left(1\right)}}+\lambda_{2}^{2p_{2}^{\left(1\right)}}+\lambda_{3}^{2p_{2}^{\left(1\right)}}-3\right)\\ & \;\;+\frac{c_{1}^{\left(2\right)}}{2{\left(p_{1}^{\left(2\right)}\right)}^{2}}\left(\alpha_{1}^{2p_{1}^{\left(2\right)}}+\alpha_{2}^{2p_{1}^{\left(2\right)}}+\alpha_{3}^{2p_{1}^{\left(2\right)}}-3\right)+\frac{1}{3}A\left[Q^{2}+{\left(\frac{Q+b}{2}\right)}^{2}+{\left(\frac{Q-b}{2}\right)}^{2}\right]\\ & \;\;-\frac{4}{9}B\left[Q^{3}+{\left(\frac{Q+b}{2}\right)}^{3}+{\left(\frac{Q-b}{2}\right)}^{3}\right]+\frac{2}{9}C\left[Q^{4}+{\left(\frac{Q+b}{2}\right)}^{4}+{\left(\frac{Q-b}{2}\right)}^{4}\right]. \end{array}$$

As the second and third directions must be stress free, the associated principal Cauchy stresses, defined by (2.10), are equal to

3.8 $$\begin{array}{rl} T_{i}^{\left(\mathrm{lce}\right)} & \;=\frac{c_{1}^{\left(1\right)}}{p_{1}^{\left(1\right)}}\left(\lambda_{i}^{2p_{1}^{\left(1\right)}}-\lambda_{2}^{2p_{1}^{\left(1\right)}}\right)+\frac{c_{2}^{\left(1\right)}}{p_{2}^{\left(1\right)}}\left(\lambda_{i}^{2p_{2}^{\left(1\right)}}-\lambda_{2}^{2p_{2}^{\left(1\right)}}\right)+\frac{c_{1}^{\left(2\right)}}{p_{1}^{\left(2\right)}}\left(\alpha_{i}^{2p_{1}^{\left(2\right)}}-\alpha_{2}^{2p_{1}^{\left(1\right)}}\right)\\ & \;=\frac{c_{1}^{\left(1\right)}}{p_{1}^{\left(1\right)}}\left(\lambda_{i}^{2p_{1}^{\left(1\right)}}-\lambda_{3}^{2p_{1}^{\left(1\right)}}\right)+\frac{c_{2}^{\left(1\right)}}{p_{2}^{\left(1\right)}}\left(\lambda_{i}^{2p_{2}^{\left(1\right)}}-\lambda_{3}^{2p_{2}^{\left(1\right)}}\right)+\frac{c_{1}^{\left(2\right)}}{p_{1}^{\left(2\right)}}\left(\alpha_{i}^{2p_{1}^{\left(2\right)}}-\alpha_{3}^{2p_{1}^{\left(1\right)}}\right),\\ & i=1,2,3. \end{array}$$

The corresponding first Piola–Kirchhoff stress are defined by (2.11).

**Figure 4.**
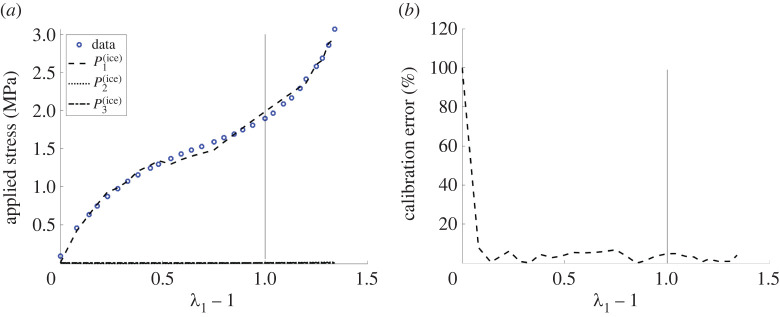
(*a*) Comparison of the computed first Piola–Kirchhoff stress $P_{1}^{\left(\mathrm{lce}\right)}$ as a function of the longitudinal strain $\lambda_{1}-1$ (dashed line), with the experimental data for the applied stress recorded in table 2 (circles); (*b*) the calibration relative error given by the absolute value of the difference between the computed and experimental values of the applied stress, divided by the experimental value. The model function is described by (3.7) with parameter values recorded in table 1 (see also fig. 8 of [6]). In each plot, the vertical line is drawn at the critical strain where the director suddenly rotates by π/2. (Online version in colour.)

Figure 4*a* shows the computed first Piola–Kirchhoff stresses in the three directions compared to the given data. Numerically, we employed a nonlinear least-squares procedure (lsqnonlin.m) implemented in Matlab [65,66], whereby the optimal model coefficients were determined by minimizing the residual between the Piola–Kirchhoff stresses in the first and second directions and the associated data values at the prescribed stretches, respectively, while the stresses in the third direction are set equal to zero. Namely, in the first direction, the applied stress data are recorded in table 2, while in the second direction the stress data are all taken to be zero. This is different from the usual model calibration in uniaxial tension where the stretches in the direction orthogonal to that of the applied force are equal and the associated stresses are guaranteed to be equal as well. The computed relative error is displayed in figure 4*b*. The resulting parameter values are provided in table 1.

**Figure 5.**
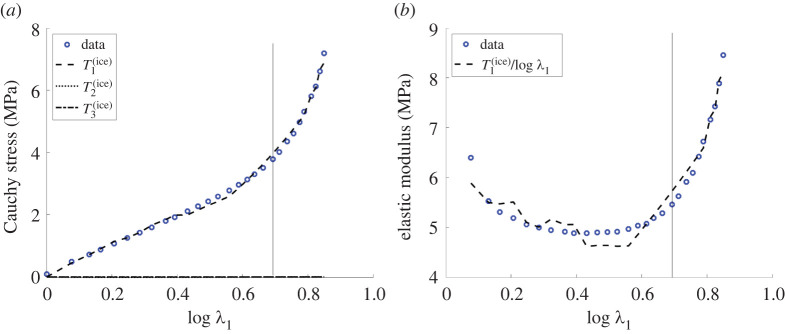
(*a*) The principal Cauchy stresses given by (3.8) and (*b*) the nonlinear elastic modulus $T_{1}^{\left(\mathrm{lce}\right)}/\log\lambda_{1}$ versus the logarithmic longitudinal strain $\log\lambda_{1}$ (see also fig. 13 of [6]). In each plot, the vertical line is drawn at the critical strain where the director suddenly rotates by π/2. (Online version in colour.)

Figure 5 illustrates the principal Cauchy stresses given by (3.8) and also the nonlinear stretch modulus $T_{1}^{\left(\mathrm{lce}\right)}/\log\lambda_{1}$ versus the logarithmic strain $\log\lambda_{1}$. Nonlinear elastic moduli for homogeneous isotropic hyperelastic materials are defined in [67]. For nematic LCEs, different elastic moduli at small strain are analysed in [56].

## 4. Conclusion

In this paper, we focused on the uniaxial deformation of a nematic LCE, which, in contrast to many other rubber-like materials and nematic elastomers, when extended, becomes thicker in a perpendicular direction if the strain is sufficiently large, while its volume remains unchanged. To capture this unusual large-strain auxetic response, we represented the material using an Ogden-type strain-energy function and calibrated its parameters to experimental data. This is a phenomenological model capable of describing the observed mechanical behaviour regardless of the molecular composition. In this sense, the model is not unique, as other continuum models may also be in agreement with the available datasets. Nevertheless, Ogden strain-energies are particularly suited to modelling complex elastic materials, such as LCEs, because of their mathematical simplicity and their clear formulation in terms of the principal stretches, which have a direct kinematic interpretation. They are also well known to easily fit any experimental data for finite deformations, with a relatively small number of terms.

Conversely, given the model parameters listed in table 1, one should be able to obtain $\lambda_{2}$, $\lambda_{3}$, Q and b as functions of the stretch ratio $\lambda_{1}$. Hence, the auxetic elastic response at large strain should be predicted. However, the inverse problem involved is highly nonlinear, and finding an effective predictive model for this complex material behaviour remains open to future investigation.

Further, the mathematical model developed here was obtained by following traditional deterministic approaches, where average data values are used. In practice, uncertainties in experimental measurements emerge from sample to sample variability, observational data, which may be sparse, indirect, and polluted by noise, and imperfect reversibility of elastic deformations, especially at large strains. The data variability can be taken into account by more sophisticated, non-deterministic models, where model parameters follow probability distributions [68,69].

Theoretical and practical challenges related to the modelling rubber-like elasticity, which apply also to LCEs, are discussed in [70]. It is concluded there that, for a theory to be helpful in explaining the elastic responses of a material, it should take into account its properties *not only in simple extension and compression, but also in other types of strain*. For the nematic elastomer considered in this paper, the mechanical properties under multiaxial deformations [22,53,71,72] deserve to be further investigated.

## Data Availability

All supporting data for this research are included in the paper.

## Appendix A. Experimental data

We present in this appendix the experimental data for the calibration of the LCE model parameters. Details of the physical set-up are discussed in [6].

**Table 2.** Experimental data (see also figs 8 and 10 of [6]).

$\lambda_{1}$ longitudinal stretch	θ director angle	Q uniaxial order parameter	b biaxial order parameter	$\lambda_{3}$ thickness	$P_{1}^{\left(\mathrm{lce}\right)}$ (MPa) applied stress
1.0000	π/2	0.5880	−0.0102	1.0000	0.0878
1.0800	π/2	0.5610	−0.0066	0.9400	0.4561
1.1400	π/2	0.5545	−0.0084	0.9000	0.6355
1.1800	π/2	0.5448	−0.0024	0.8800	0.7453
1.2300	π/2	0.5322	−0.0108	0.8600	0.8730
1.2800	π/2	0.4866	−0.0132	0.8300	0.9757
1.3300	π/2	0.4549	−0.0156	0.8100	1.0724
1.3800	π/2	0.4298	−0.0210	0.8000	1.1540
1.4400	π/2	0.3920	−0.0228	0.7800	1.2454
1.4800	π/2	0.3885	−0.0168	0.7700	1.2951
1.5400	π/2	0.3700	−0.0270	0.7400	1.3712
1.5900	π/2	0.3421	−0.0336	0.7300	1.4298
1.6400	π/2	0.2786	−0.0534	0.7200	1.4818
1.6900	π/2	0.2500	−0.0792	0.7100	1.5269
1.7500	π/2	0.2284	−0.0870	0.7000	1.5904
1.8000	π/2	0.1699	−0.0900	0.7000	1.6464
1.8500	π/2	0.1228	−0.0948	0.7000	1.6887
1.8900	π/2	0.1053	−0.0990	0.7000	1.7487
1.9400	π/2	0.0751	−0.1128	0.7000	1.8058
2.0000	0	0.0279	−0.1470	0.7000	1.8935
2.0400	0	0.0020	−0.1518	0.7000	1.9664
2.0900	0	0.0010	−0.1128	0.7000	2.0851
2.1300	0	0.0025	−0.1080	0.7000	2.1646
2.1700	0	0.0035	−0.1050	0.7000	2.2921
2.2000	0	0.0100	−0.1008	0.7000	2.4106
2.2500	0	0.0277	−0.1002	0.7100	2.5801
2.2800	0	0.0400	−0.1020	0.7100	2.6852
2.3100	0	0.0925	−0.1038	0.7200	2.8569
2.3400	0	0.1000	−0.1056	0.7200	3.0704
